# LGBTQ+ individuals are not explicitly represented in emergency medicine simulation curricula

**DOI:** 10.12688/mep.20242.1

**Published:** 2024-04-30

**Authors:** Jessica Bod, Samuel Buck, Iris Chandler, Katja Goldflam, Alina Tsyrulnik, Ryan Coughlin, Jessica Fujimoto, Melissa Joseph, David Della-Giustina, Manali Phadke, Dowin Boatright

**Affiliations:** 1 Emergency Medicine, Yale University, New Haven, Connecticut, USA; 2 Emergency Medicine, UCSF Fresno, Fresno, CA, USA; 3 Emergency Medicine, New York University, New York, New York, USA

**Keywords:** Emergency Medicine, Simulation, LGBTQ+

## Abstract

**Background:**

Medical educational societies have emphasized the inclusion of marginalized populations, including the lesbian, gay, bisexual, transgender and queer (LGBTQ+) population, in educational curricula. Lack of inclusion can contribute to health inequality and mistreatment due to unconscious bias. Little didactic time is spent on the care of LGBTQ+ individuals in emergency medicine (EM) curricula. Simulation based medical education can be a helpful pedagogy in teaching cross-cultural care and communication skills. In this study, we sought to determine the representation of the LGBTQ+ population in EM simulation curricula. We also sought to determine if representations of the LGBTQ+ population depicted stigmatized behavior.

**Methods:**

We reviewed 971 scenarios from six simulation case banks for LGBTQ+ representation. Frequency distributions were determined for major demographic variables. Chi-Squared or Fisher’s Exact Test, depending on the cell counts, were used to determine if relationships existed between LGBTQ+ representation and bank type, author type, and stigmatized behavior.

**Results:**

Of the 971 scenarios reviewed, eight (0.82%) scenarios explicitly represented LGBTQ+ patients, 319 (32.85%) represented heterosexual patients, and the remaining 644 (66.32%) did not specify these patient characteristics. All cases representing LGBTQ+ patients were found in institutional case banks. Three of the eight cases depicted stigmatized behavior.

**Conclusions:**

LGBTQ+ individuals are not typically explicitly represented in EM simulation curricula. LGBTQ+ individuals should be more explicitly represented to reduce stigma, allow EM trainees to practice using gender affirming language, address health conditions affecting the LGBTQ+ population, and address possible bias when treating LGBTQ+ patients.

## Introduction

Over the past decade, medical education societies have called for the development of curricula to address the care of marginalized populations, including patients identifying as part of the lesbian, gay, bisexual, transgender, and queer (LGBTQ+) community ^1– 4^. The LGBTQ+ community faces unique challenges when interacting with the healthcare system, including overt discrimination, implicit bias, microaggressions and disparities in mental and physical health ^5, 6^. Moreover, LGBTQ+ medical students are subject to higher levels of mistreatment during their training than their straight counterparts ^7^.

Racial/ethnic and sex/gender curricular biases are well documented ^8– 12^. These biases include omission of people of color and women from curricula, imbalance in the presentation of issues, and ignoring inequalities in health and illness ^9^. Underrepresentation of LGBTQ+ individuals and families in educational materials can contribute to unconscious biases impacting both patients and medical trainees by centralizing heteronormativity and contributing to the isolation and “invisibility” of LGBTQ+ individuals ^8, 9^. When the LGBTQ+ population is represented in case-based curricula, there is a documented trend to perpetuate stigma by emphasizing associations between the LGBTQ+ community and sexually transmitted illnesses such as HIV/AIDS ^8^.

Medical education initiatives have been instrumental in advocating for the equitable treatment of LGBTQ+ patients and inclusivity in medical training environments ^2^. Despite significant progress, the median time spent addressing LGBTQ+ health in United States and Canadian medical schools is only five hours across the entire curriculum ^13^. Further, two-thirds of medical students rate their LGBTQ+-related curricula as fair, poor, or very poor ^14^. A 2014 survey of emergency medicine (EM) residencies found that only 33% of programs incorporated topics of LGBTQ+ health in their curricula and spent an average of 45 minutes on this subject ^15^.

Simulation-based medical education (SBME) can be a powerful way to incorporate LGBTQ+ healthcare and inclusivity in both undergraduate and graduate medical education. SBME fosters active learning while allowing trainees to practice skills and learn from mistakes without risk of adverse effects on patients ^16^. EM residency curricula place an increasing emphasis on simulation as a pedagogic tool ^17^, and the Accreditation Council for Graduate Medical Education (ACGME) has endorsed simulation as an important modality to teach patient care, medical knowledge, interpersonal and communication skills, and professionalism ^18^. Interactive workshops and simulations can improve the delivery of cross-cultural healthcare ^19, 20^, and can increase the knowledge base related to the care of transgender patients ^21, 22^.

In this study, we sought to assess the representation of the LGBTQ+ population in EM simulation curricula. Specifically, we were interested in the extent to which EM simulation scenarios are explicitly inclusive of the LGBTQ+ population and whether the inclusion of LGBTQ+ individuals in simulated patient encounters centers around stigmatized diagnoses and behaviors.

## Methods

The study authors reviewed 971 unique scenarios from six different simulation case banks. Three case collections were obtained from publicly available educational resources: MedEdPortal ^23^, the Council of Residency Directors in EM (CORD) simulation case bank ^24^ and EM Sim Cases ^25^. These resources were chosen for their popularity, ^26^ endorsement by professional societies ^24^, and use of peer review. The MedEdPortal cases were identified by performing an advanced search of titles containing the words “emergency medicine” and “simulation” in the summer of 2021. In addition to the national resources, we were able to access a convenience sample of three private institutional case banks associated with residency training programs, one located in the northeast region of the USA and two on the west coast. Each of these programs has a large simulation training center. These included banks from two four-year programs and one three-year program. Permission to use the banks was obtained from the relevant organizations, departments, and institutions prior to analysis.

The simulation cases in each case bank were reviewed for LGBTQ+ representation. Coding guidelines were delineated prospectively in a data dictionary and the authors arbitrated collaboratively any ambiguity or question pertaining to the data dictionary until consensus was achieved prior to any data analysis. LGBTQ+ representation in simulated scenarios can be explicit delineation of patient sexual or gender orientation in case scripts such as “the patient is a male to female transwoman,” or implicitly in depicting a romantic or familial relationship such as “the patient asks the trainee to notify his husband.” In some instances, particularly in pediatric scenarios, sexual diversity can be represented by family members accompanying the patient. For example, “the patient is a six-week-old male accompanied by his mothers.” Accordingly, demographic and behavioral variables coded were patient sexual orientation, relationship type depicted, gender identity, diagnosis, and behavioral concerns (such as intoxication, violence, or multiple sexual partners). When no sexual orientation or relationship was specified, the scenario was coded as “not specified.” In contrast, when gender identity was not specified, the authors assumed a cis-gender patient. This assumption was based on common descriptions of patients in the English language in which cis-gender identity is not typically specified in medical case presentations though the cis-gender identity is implicit whereas trans identities are typically explicitly stated. Each case was coded by a single reviewer and arbitrated with another reviewer in cases with unclear language. All scenarios identified as depicting an LGBTQ+ patient or relationship were analyzed for stereotyping or stigmatizing diagnoses and behaviors.

The primary outcome measure was the number of simulation cases explicitly representing the LGBTQ+ population. Additional outcomes included assessment of whether cases representing the LGBTQ+ population depicted stigmatized diagnoses or behaviors.

Frequency distributions were determined for major demographic variables. Chi-Squared or Fisher’s Exact Test, depending on the cell counts, were used to determine if relationships existed between LGBTQ+ representation and bank type, author type, and behavioral concerns and p<.05 was deemed significant. This study was reviewed by our institutional review board and was determined to not involve human subjects. 

## Results

Of the 971 scenarios reviewed, eight (0.82%) scenarios explicitly represented LGBTQ+ patients, 319 (32.85%) specified or implied through family affiliations that the patient was heterosexual, and the remaining 644 (66.32%) did not specify these patient characteristics ^27^. No additional LGBTQ+ representation was identified when looking at other relationships depicted in scenarios, such as the parents of simulated pediatric patients. Scenario characteristics including LGBTQ+ representation, bank type, and case author type are summarized in Table 1.

**Table 1.** Simulation case characteristics.

	n	percent
**Patient Representation**		
Unspecified Patient	644	66.32
Heterosexual Patient	319	32.85
LGBTQ+ Patient	8	0.82
**Case Bank Type**		
Private Institutional Bank	655	67.45
Publicly Available Bank	316	32.54
**Author Type**		
Faculty Author	361	37.18
Resident Author	356	36.66
Unknown Author	228	23.48
Multiple Authors/Other	15	1.54
Medical Student Author	11	0.13

Of the eight scenarios representing LGBTQ+ patients, seven specifically represented gay men and one represented a male bisexual patient. No lesbian or transgender patients were specifically represented. The diagnoses and behavioral descriptions of the eight scenarios representing LGBTQ+ patients are specified in Table 2. All eight cases representing LGBTQ+ patients were found in institutional case banks (p=0.0035). There were no significant relationships between LGBTQ+ representation and author type.

**Table 2.** Cases representing LGBTQ+ patients.

	Patient	Clinical Case	Behavioral Concerns
Case 1	Gay Man	Abdominal Compartment Syndrome	None
Case 2	Gay Man	Cement in Eye	Opiate Use Disorder
Case 3	Bisexual	Disseminated TB	Multiple Sex Partners
Case 4	Gay Man	PRES	None
Case 5	Gay Man	End of Life/Palliative Care	None
Case 6	Gay Man	Lemierre's Syndrome	Multiple Sex Partners
Case 7	Gay Man	Appendicitis	None
Case 8	Gay Man	Fatigue	None

## Discussion

Although medical educational societies stress the importance of responsiveness to diverse patient populations in EM medical education attention to the LGBTQ+ population in EM training curricula remains limited ^14, 15^. The ACGME has determined that respect and responsiveness to diverse populations is a core competency amongst all physicians and must be integrated into training curricula ^3^. Survey data, which likely underrepresents the true LGBTQ+ population, suggests that greater than 7% of the US population belongs to this group ^28^. In our analysis, only 0.82% of cases explicitly represented LGBTQ+ individuals illustrating that LGBTQ+ individuals are not typically intentionally represented in the EM simulation curricula reviewed.

Lesbians and transgender individuals are not represented at all in our data set. Despite decades of calls for inclusion of LGBTQ+ issues in medical curricula, and the effectiveness of simulation in teaching cross-cultural care, writers at all levels of training are not writing this population into their scripts. Transgender health is an especially neglected topic.

The majority of scenarios reviewed did not specify or imply the sexual orientation or gender identity of simulated patients at all. As SBME continues to be an integral part of EM education, lack of representation of the LGBTQ+ population in simulated patient encounters has the potential to reinforce unconscious assumptions of heterosexual normativity and to reinforce the invisibility felt by LGBTQ+ individuals in healthcare and medical training ^5– 9^. Our results indicate that there is an opportunity for future authors to intentionally represent the LGBTQ+ population and to incorporate inclusivity in the structure of SMBE. Other structural components of EM education such as case conferences and question banks may be a key area of future investigation into ways to improve LGBTQ+ inclusivity in EM training.

The number of cases explicitly representing the LGBTQ+ population was not sufficient to determine whether this population is being used to depict stigmatized behaviors. Additional work with a larger sample size would be necessary to determine if stigmatized behaviors and diagnoses such as drug use and sexually transmitted illnesses are associated with LGBTQ+ representation.

Our analysis illustrates that LGBTQ+ individuals are not typically explicitly represented in EM simulation curricula. We recommend that EM curricular directors recognize the importance of simulation in teaching issues related LGBTQ+ health to trainees. We suggest that simulation scenarios should be written to allow trainees to practice using gender affirming language, recognize and treat health conditions affecting the LGBTQ+ population, and address possible bias when treating LGBTQ+ patients. Particular attention should be given to creating simulated encounters focused on transgender healthcare, as the use of gender affirming medications and procedures can have important implications when transgender patients present to the emergency department. Finally, we recommend that more attention be paid to representing LGBTQ+ individuals across a variety of simulated encounters in order to decrease the stigma associated with implied heteronormativity and decrease the invisibility felt by LGBTQ+ trainees.

Our study is limited by the use of simulation databases that we were able to access, particularly when considering the vast number of private institutional simulation banks. This introduces selection bias. There may be more inclusive simulation banks we were unaware of. Simulation case banks are inherently variable, are not standardized and may be modified over time. Further, because of the scant number of scenarios in which LGBTQ+ identity is specified, we are not able to draw conclusions about whether these cases disproportionately depict stigmatized diagnoses or behaviors. Finally, because not all scenarios explicitly specify the sexual or gender identity of the patients, we make assumptions about implied heteronormativity in drawing our conclusions.

## Conclusions

LGBTQ+ individuals are not typically explicitly represented in EM simulation curricula. LGBTQ+ individuals should be more explicitly represented to reduce stigma, allow EM trainees to practice using gender affirming language, address health conditions affecting the LGBTQ+ population, and address possible bias when treating LGBTQ+ patients. More study is needed to determine if SMBE cases representing the LGBTQ+ population disproportionally depicts stigmatized behaviors and diagnoses.

## Data Availability

Dataverse: LGBTQ+ Individuals are not Explicitly Represented in Emergency Medicine Simulation Curricula. https://doi.org/10.7910/DVN/KY4TF0 ^27^. This project contains the following underlying data: Simulation Diversity Project.xlsx (This file contains the simulation cases from all databases that were reviewed for the study. The characteristics of the relationships and gender identities depicted as well as the clinical problem and any stigmatizing behaviors are described.) Data are available under the terms of the Creative Commons Zero “No rights reserved” data waiver (CC0 1.0 Public domain dedication).

## References

[1] Daniel H Butkus R : Lesbian, gay, bisexual, and transgender health disparities: executive summary of a policy position paper from the American college of physicians. *Ann Intern Med.* 2015;163(2):135–7. 10.7326/M14-2482 25961598

[2] Association of American Medical Colleges: Implementing curricular and institutional climate changes to improve health care for individuals who are LGBT, gender nonconforming, or born with DSD: a resource for medical educators.Washington, D. C.: AAMC;2014. Reference Source

[3] Accreditation Council for Graduate Medical Education: ACGME common program requirements. ACGME;2022. Reference Source

[4] Bohnert CA Combs RM Noonan EJ : Gender minorities in simulation: a mixed methods study of medical school Standardized Patient programs in the United States and Canada. *Simul Healthc.* 2021;16(6):e151–e158. 10.1097/SIH.0000000000000532 33273422

[5] Institute of Medicine (US) Committee on Lesbian, Gay, Bisexual, and Transgender Health Issues and Research Gaps and Opportunities: The health of lesbian, gay, bisexual, and transgender people: building a foundation for better understanding. Washington D.C.: National Academies Press;2011. 10.17226/13128 22013611

[6] Institute of Medicine (US) Board on the Health of Select Populations: Collecting sexual orientation and gender identity data in electronic health records: workshop summary. Washington D.C.: National Academies Press;2013. 10.17226/18260 23967501

[7] Hill KA Samuels EA Gross CP : Assessment of the prevalence of medical student mistreatment by sex, race/ethnicity, and sexual orientation. *JAMA Intern Med.* 2020;180(5):653–665. 10.1001/jamainternmed.2020.0030 32091540 PMC7042809

[8] Tubres S Krebs E Axtell S : The hidden curriculum in multicultural medical education: the role of case examples. *Acad Med.* 2002;77(3):209–216. 10.1097/00001888-200203000-00007 11891157

[9] Martin GC Kirgis J Sid E : Equitable imagery in the preclinical medical school curriculum: findings from one medical school. *Acad Med.* 2016;91(7):1002–1006. 10.1097/ACM.0000000000001105 26839941

[10] Dijkstra AF Verdonk P Lagro-Janssen AL : Gender bias in medical textbooks: examples from coronary heart disease, depression, alcohol abuse and pharmacology. *Med Educ.* 2008;42(10):1021–1028. 10.1111/j.1365-2923.2008.03150.x 18761614

[11] Murray-García JL García JA : The institutional context of multicultural education: what is *your* institutional curriculum? *Acad Med.* 2008;83(7):646–652. 10.1097/ACM.0b013e3181782ed6 18580080

[12] Amutah C Greenidge K Mante A : Misrepresenting race - the role of medical schools in propagating physician bias. *N Engl J Med.* 2021;384(9):872–878. 10.1056/NEJMms2025768 33406326

[13] Obedin-Maliver J Goldsmith ES Steward L : Lesbian, gay, bisexual, and transgender-related content in undergraduate medical education. *JAMA.* 2011;306(9):971–977. 10.1001/jama.2011.1255 21900137

[14] White W Brenman S Paradis E : Lesbian, gay, bisexual, and transgender patient care: medical students’ preparedness and comfort. *Teach Learn Med.* 2015;27(3):254–263. 10.1080/10401334.2015.1044656 26158327

[15] Moll J Krieger P Moreno-Walton L : The prevalence of lesbian, gay, bisexual, and transgender health education and training in emergency medicine residency programs: what do we know? *Acad Emerg Med.* 2014;21(5):608–611. 10.1111/acem.12368 24842513

[16] Ziv A Ben-David S Ziv M : Simulation based medical education: an opportunity to learn from errors. *Med Teach.* 2005;27(3):193–199. 10.1080/01421590500126718 16011941

[17] Okuda Y Bond W Bonfante G : National growth in simulation training within emergency medicine residency programs, 2003–2008. *Acad Emerg Med.* 2008;15(11):1113–1116. 10.1111/j.1553-2712.2008.00195.x 18717652

[18] Ten Eyck RP : Simulation in emergency medicine training. *Pediatr Emerg Care.* 2011;27(4):333–344; quiz 342–4. 10.1097/PEC.0b013e3182131fe0 21467889

[19] Beauchamp GA McGregor AJ Choo EK : Incorporating sex and gender into culturally competent simulation in medical education. *J Womens Health (Larchmt).* 2019;28(12):1762–1767. 10.1089/jwh.2018.7271 30601088

[20] Ozkara San E : Development of the diverse standardized patient simulation cultural competence education strategy. *Nurs Educ Perspect.* 2019;40(6):E31–E33. 10.1097/01.NEP.0000000000000519 31206417

[21] McCave EL Aptaker D Hartmann KD : Promoting affirmative transgender health care practice within hospitals: an IPE standardized patient simulation for graduate health care learners. *MedEdPORTAL.* 2019;15: 10861. 10.15766/mep_2374-8265.10861 32051844 PMC7010321

[22] Greene RE Blasdel G Cook TE : How do OSCE cases activate learners about transgender health? *Acad Med.* 2020;95(12S):S156–S162. 10.1097/ACM.0000000000003704 32889930

[23] www.mededportal.comAccessed March 31, 2021.

[24] CORD Oral Board and Simulation Cases. Council of emergency medicine residency directors. Accessed April 15, 2021. Reference Source

[25] www.emsimcases.comAccessed on April 7, 2021.

[26] Caners K Baylis J Heyd C : Sharing is caring: how EM Sim Cases (EMSimCases.com) has created a collaborative simulation education culture in Canada. *CJEM.* 2020;22(6):819–821. 10.1017/cem.2020.392 35114788

[27] Bod J : LGBTQ+ individuals are not explicitly represented in emergency medicine simulation curricula.[Dataset], Harvard Dataverse, V12024. 10.7910/DVN/KY4TF0 PMC11200058 38932993

[28] Gallup Organization: Americans’ self-identified sexual orientation.Washington D.C.: Gallup Organization,2022.

