# Supplementary material for: LGBTQ+ individuals are not explicitly represented in emergency medicine simulation curricula
Source: MedEdPublish (2016). 2024 Apr 30;14:30. [Version 1] doi: 10.12688/mep.20242.1 (PMC11200058; doi:10.12688/mep.20242.1)
Supplement: Peer review history

# Reviewer report: Reviewer response for version 1

**DOI:** 10.21956/mep.21670.r37371

**Published:** 2024-06-25

**Authors:** Michael I. Kruse

**Affiliations:** 1 McMaster University, Hamilton, Ontario, Canada

Overall, a good solid paper that answers an important question. The methods are sound, though under-reported (see below). The results and conclusions are appropriate given the findings of the study.

**Introduction**

 1. Graph 3, line4-6: Cites out of date paper, Moll et al updated their study in 2021 [Refer ref 1]. With a 43% response rate, 75% of programs offered education content on LGBTQ health. This shows improvement over the previous study. Can be included to make the intro more relevant without hindering the purpose of the study.

**Methods**

 1. Good description of the data dictionary development, though the language was a bit dense and can be simplified somewhat. E.g. graph 2, line 1-3 is unnecessarily dense as it essentially describes the collaborative development of a data dictionary through iterative consensus. The data dictionary is not available in the appendix and would be needed to replicate this study fully.

 2. Graph 2, last line: there is no description of how the analysis for stereotyping or stigmatizing diagnoses was to be done. Though there was not enough data to allow for this analysis, it would be helpful is a brief description of these methods would be included for reproducibility in other data sets.

 3. Graph 4: can the authors expand on the choice of statistical test beyond "depending on cell counts". This is an appropriate test to compare data set distribution, but the choice of test was unclear. I think I know what the authors were getting at, but a clear description of how the tests would be chosen would be helpful for reproduction.

**Results**

 1. Well reported, clear and concise.

 2. There is no mention of the statistical findings for a relationship between bank type and behavioral concerns as outlined in the methods, only commented on the author. This is commented on in the discussion but not presented in results.

 3.There is also no mention of what statistical method was chosen, as it was either going to be Chi Squared or Fisher Exact. Even if null result, can this be reported for completeness?

**Discussion**

 1. Graph 1 line 1 and 2 - there is a missing comma after "EM medical education". Sentence is hard to read as a result.

 2. Graph 1 line 5: The citation only lists sexual orientation prevalence and transgender and gender diverse populations would be helpful to highlight, especially since they were not found in any of the simulations and the authors comment on this important need. Herman et al,2022 [Ref 2] and Meerwijk and Sevelius [Ref 3] are 2 possibilities.

 3. Fully support recommendations, great list. May include a research oriented recommendation to better characterize stigma in simulated encounters with LGBTQ populations. This is included in the conclusions but not discussions.

Have any limitations of the research been acknowledged?

Yes

Is the study design appropriate and does the work have academic merit?

Yes

Is the work clearly and accurately presented and does it cite the current literature?

Partly

If applicable, is the statistical analysis and its interpretation appropriate?

Yes

Are all the source data underlying the results available to ensure full reproducibility?

Yes

Are the conclusions drawn adequately supported by the results?

Yes

Are sufficient details of methods and analysis provided to allow replication by others?

Partly

Reviewer Expertise:

Care of sexual and gender minorities in the emergency department and pre-hospital environment.

I confirm that I have read this submission and believe that I have an appropriate level of expertise to confirm that it is of an acceptable scientific standard, however I have significant reservations, as outlined above.

## References

[1] : The prevalence of lesbian, gay, bisexual, and transgender health education and training in emergency medicine residency programs: Where are we now?. *AEM Educ Train* .2021;5(2) : 10.1002/aet2.10580 e10580 10.1002/aet2.10580 33817541 PMC8011615

[2] : How Many Adults and Youth Identify as Transgender in the United States?. *The Williams Institute, UCLA School of Law* .2022;

[3] : Transgender Population Size in the United States: a Meta-Regression of Population-Based Probability Samples. *Am J Public Health* .2017;107(2) : 10.2105/AJPH.2016.303578 e1-e8 10.2105/AJPH.2016.303578 28075632 PMC5227946

# Reviewer report: Reviewer response for version 1

**DOI:** 10.21956/mep.21670.r37366

**Published:** 2024-06-25

**Authors:** Daniel J. Egan

**Affiliations:** 1 Harvard Affiliated Emergency Medicine Residency, Boston, Massachusetts, USA

Thanks for your work on this important topic. This study represents an analysis of publicly available simulation cases in emergency medicine as well as a small cohort of institutional-based curricula. The largest limitation of the study is the fact that we do not know what is happening locally in residency programs who may be more inclusive and not publishing their simulation curricula. I suspect there are more sites where this is included, but not published given the lack of available locations to publish these type of cases on a regular basis. The CORD database is somewhat outdated and likely not having regular updates. It would be helpful in all of your simulation cases you evaluated to identify when they were submitted if possible given the more recent attention to themes of diversity and inclusive language. I think that your conclusion might be slightly overstated by making the generalization that the LGBTQ population is not represented in curricula. I think you need to temper this slightly to clarify that this is only in the cases to which you had access.

 In your introduction section, there is a more recent reference related to the curricula of LGBTQ+ health that was updated by the same author group you cite as reference #15 which has a 2020 survey result comparing the change over time (Refer ref [1]). Would recommend that you update this to reflect this more recent data. You could also consider

 Regarding your method of identifying stereotypic or stigmatizing diagnoses and behaviors, how did your group come to consensus on what would be identified? I wonder if you considered having two reviewers given that this might be less obvious than the explicit statements on gender or sexual orientation? Additionally, you could consider a spot check of a second reviewer in order to demonstrate the validity of the abstraction. 

 The discussion section is particularly strong as it relates to invisibility and the support of heteronormative assumptions. While you did the analysis forward (meaning looked at identification of LGBTQ+ people and behaviors), it would be curious if there were cases specific to some of the more biased behaviors you mention. For example, were there a number of HIV/AIDS or STI cases in the simulation cases you reviewed that did *not* have the patient identifying as a gay or bisexual man? This might be an interesting alternative approach to suggest that perhaps there was intentionality in not using this group to represent the disease which one could argue may be a step in the right direction.

Have any limitations of the research been acknowledged?

Yes

Is the study design appropriate and does the work have academic merit?

Yes

Is the work clearly and accurately presented and does it cite the current literature?

Yes

If applicable, is the statistical analysis and its interpretation appropriate?

Yes

Are all the source data underlying the results available to ensure full reproducibility?

Partly

Are the conclusions drawn adequately supported by the results?

Yes

Are sufficient details of methods and analysis provided to allow replication by others?

Yes

Reviewer Expertise:

Medical education, LGBT health

I confirm that I have read this submission and believe that I have an appropriate level of expertise to confirm that it is of an acceptable scientific standard.

## References

[1] : The prevalence of lesbian, gay, bisexual, and transgender health education and training in emergency medicine residency programs: Where are we now?. *AEM Educ Train* .2021;5(2) : 10.1002/aet2.10580 e10580 10.1002/aet2.10580 33817541 PMC8011615

# Reviewer report: Reviewer response for version 1

**DOI:** 10.21956/mep.21670.r37364

**Published:** 2024-06-24

**Authors:** Dani Hall

**Affiliations:** 1 University College Dublin, Dublin, Ireland

This is an interesting report, highlighting a gap in LGBTQ+ health and affirmative language in SBME for EM trainees in the US.

**Results**

 There are a couple of additional results you may have collected that would be interesting to share:

 1. Did any of the 8 simulations (adult and paediatric) that included LGBTQ+ simulated patients include any intended learning outcomes / areas for debrief around LGBTQ+ affirmative, non-judgemental, non-heteronormative language?

 2. Did any of the 8 LGBTQ+ sims describe co-design?

 3. You mention statistical testing and p values but these are not described in your tables. Can you share additional detail on these.

 Table 2 should be expanded to incorporate all this information.

**Literature cited**

 A couple of additional references you may want to include are: Refer references [1,2,3].

Have any limitations of the research been acknowledged?

Yes

Is the study design appropriate and does the work have academic merit?

Yes

Is the work clearly and accurately presented and does it cite the current literature?

Partly

If applicable, is the statistical analysis and its interpretation appropriate?

Partly

Are all the source data underlying the results available to ensure full reproducibility?

No source data required

Are the conclusions drawn adequately supported by the results?

Yes

Are sufficient details of methods and analysis provided to allow replication by others?

Yes

Reviewer Expertise:

LGBTQ+ education for paediatric staff

I confirm that I have read this submission and believe that I have an appropriate level of expertise to confirm that it is of an acceptable scientific standard, however I have significant reservations, as outlined above.

## References

[1] : Gender Diverse Representation in Patient Simulation: A Scoping Review. *Acad Med* .2022;97(11S) : 10.1097/ACM.0000000000004926 S107-S116 10.1097/ACM.0000000000004926 35947464

[2] : Demonstrating LGBTQ+ Affirmative Practice in Groups:: Developing Competence through Simulation-Based Learning. *Clin Soc Work J* .2022;50(3) : 10.1007/s10615-022-00850-2 297-307 10.1007/s10615-022-00850-2 36034182 PMC9395481

[3] : Closing the gap: Using simulation to improve public health clinicians’ affirming beliefs and behaviors with LGBT clients. *Clinical Simulation in Nursing* .2022;71: 10.1016/j.ecns.2022.03.005 128-134 10.1016/j.ecns.2022.03.005
